# Anti-Inflammatory and Antimicrobial Effects of *Eucalyptus* spp. Essential Oils: A Potential Valuable Use for an Industry Byproduct

**DOI:** 10.1155/2023/2582698

**Published:** 2023-06-28

**Authors:** Emilly S. Salvatori, Letícia V. Morgan, Samara Ferrarini, Gabriela A. L. Zilli, Adriano Rosina, Manuelle O. P. Almeida, Helen C. S. Hackbart, Renan S. Rezende, Daniel Albeny-Simões, J. Vladimir Oliveira, Adriana Gasparetto, Liz G. Müller, Jacir Dal Magro

**Affiliations:** ^1^School of Health, Community University of Chapecó Region (Unochapecó), Chapecó, SC, Brazil; ^2^Graduate Program in Environmental Sciences, Community University of Chapecó Region (Unochapecó), Chapecó, SC, Brazil; ^3^School of Agriculture and Environment, Community University of Chapecó Region (Unochapecó), Chapecó, SC, Brazil; ^4^Graduate Program in Food Science Technology, UFPel, Pelotas, RS, Brazil; ^5^Department of Chemical and Food Engineering, UFSC, Florianópolis, SC, Brazil

## Abstract

In Brazil, the use of *Eucalyptus* is focused on the production of wood or pulp for the paper industry but without any general recovery of waste, with leaves and branches being left on the ground. One possibility is to use these residues as raw materials in the production of industrially relevant and value-added compounds such as essential oil. The aim of the present study was to investigate the chemical composition, yield, anti-inflammatory/antinociceptive activities, and acute toxicity in mice, as well as the antimicrobial effects of essential oils from the leaves of 7 varieties of *Eucalyptus* and hybrids against *Escherichia coli*, *Staphylococcus aureus,* and *Candida albicans*. The extraction of oils was carried out using hydrodistillation, and they were analyzed by gas chromatography coupled to mass spectrometry. Urocam and Grancam were the plants that obtained the highest oil yield, with yields of 3.32 and 2.30%, respectively. The main chemical components identified in these plants were 1.8 cineole and *α*-pinene. The antinociceptive effect of the 7 oils (50 mg/kg, p.o.) was initially assessed in the acetic acid-induced writhing test. In this assay, a significant (*p* < 0.05) antinociceptive/anti-inflammatory effect was observed from 4 tested essential oils (*E. benthamii, E. saligna,* and the hybrids Urocam and Grancam) when compared to the vehicle-treated group. This effect was then confirmed in the formalin-induced paw licking test. No toxicological effects or alterations were observed in motor coordination after the administration of the studied oils to the animals. In the antimicrobial evaluation, the seven essential oils inhibited the growth of *S. aureus, E. coli*, and *C. albicans* at different concentrations. Collectively, these results demonstrate that the essential oil from the leaves and branches of *Eucalyptus* species and varieties present potential biomedical applications and represent a source of antimicrobial and/or anti-inflammatory compounds.

## 1. Introduction

*Eucalyptus* species (Myrtaceae) are native to Australia, New Zealand, and Tasmania [1] and are well known for their economic importance in wood and paper production (forest industry) [2, 3]. *Eucalyptus* plantations occupy 5.7 million hectares of planted trees in Brazil [4], and the main destination of these plants is the forestry industry, generating economic benefits but, on the other hand, millions of tons of waste, which result in considerable costs for the industry and the environment [3].

In Brazil, the use of *Eucalyptus* is focused on the production of wood or pulp for the paper industry [5], but without any general recovery of waste, with leaves and branches being left on the ground. One possibility is to use these residues as raw materials in the production of industrially relevant and value-added compounds such as essential oil [6]. In fact, *Eucalyptus* essential oil is widely used in industry, especially in pharmaceuticals and perfumery. *Eucalyptus* is a heteroblastic species, and genetic variations have been detected both within and between populations at the juvenile coppice and adult leaf stages, suggesting that the populations may involve quite different ontogenetic trajectories [7].

Some substances of therapeutic interest found in *Eucalyptus* essential oils are *α*-pinene, *α*-phellandrene, cineole, limonene, and terpineol [8, 9]. According to Piccinelli et al. [10], limonene presents antidepressant, antinociceptive effects (in neuropathic pain), and anti-inflammatory potential. Eucalyptol (cineole) inhibits tumor necrosis factor (TNF), interleukin-1, leukotrienes and thromboxane in inflammatory cells, responsible for the activation of the inflammation process [11]. Bayala et al. [12] demonstrated that *α*-pinene, limonene, and cineole also exhibit satisfactory anti-inflammatory activity.

Currently, nonsteroidal anti-inflammatory drugs (NSAIDs) and glucocorticoids are the first-line drugs used to reduce the harmful events associated with inflammation [13]. These drugs exhibit important side effects, ranging from gastric irritation and ulcers to liver toxicity and chronic renal failure [14]. The scarce options for safe drugs for the treatment of chronic inflammatory diseases, such as arthritis, have led to the discovery of new medicinal agents derived from plants [15]. Thus, the disadvantages of using NSAIDs can be minimized by replacing them with safer and more efficient compounds derived from medicinal plants [15].

Essential oils from *Eucalyptus* species have been reported to have antimicrobial activity against Gram-negative (*Escherichia coli*) and Gram-positive (*Staphylococcus aureus*) bacteria, indicating that the oil can be studied as a possible natural antibiotic for the treatment of various infectious diseases caused by these two bacteria [16]. Such investigations suggest that *Eucalyptus* major compound, 1,8-cineole, may be responsible for this action, highlighting the importance of the discovery of new substances with antimicrobial potential. Microbial resistance has been growing and rendering treatment of infections difficult with the therapeutic options available; therefore, the research and development of new substances with antimicrobial potential are of primary importance [17].

Literature appraisal on the subject demonstrates the importance of preclinical investigation of the possible anti-inflammatory, antinociceptive, and antimicrobial action of different essential oils from the leaves of *Eucalyptus* species (*Eucalyptus benthamii* Maiden & Cambage, *Eucalyptus dunnii* Maiden, *Eucalyptus saligna* Smith, and *Eucalyptus grandis* Hill & Maiden) and hybrids (Urograndis − *E. grandis* × *Eucalyptus urophylla* ST Blake; Grancam − *E. grandis* × *Eucalyptus camaldulensis* Dehnh; Urocam − *E. urophylla* × *E. camaldulensis*), which are abundantly cultivated in Brazil and had never been studied regarding these biological activities [18].

Considering the abovementioned scenario, the exploration of the biological and chemical properties of plant residues from the paper industry could represent a valuable approach for these by-products. Therefore, the aim of this study was to evaluate the antinociceptive, anti-inflammatory, and antimicrobial properties of the essential oils extracted from the leaves of seven varieties of *Eucalyptus*, as well as to determine their yield and chemical composition.

## 2. Materials and Methods

### 2.1. *Eucalyptus* Sampling, Essential Oil Extraction, and Chemical Analysis

Adult *Eucalyptus* trees from four species -*Eucalyptus benthamii* Maiden & Cambage, *Eucalyptus dunnii* Maiden, *Eucalyptus saligna* Smith, and *Eucalyptus grandis* Hill&Maiden - and three hybrids: Urograndis (*E. grandis* × *Eucalyptus urophylla* ST Blake), Grancam (*E. grandis* × *Eucalyptus camaldulensis* Dehnh), and Urocam (*E. urophylla* × *E. camaldulensis*) were collected at the Agricultural Research Company and Rural Extension of Santa Catarina experimental area located in Guantambú, Santa Catarina state, Brazil (27°07055S, 52°44′04″W). The essential oils were obtained by steam distillation using 200 g fresh plant leaves over 120 min in four repetitions [19]. The yield % (wt/wt, based on the fresh weight of the mature leaves) of the extracted oil from each sample was determined by the following equation:

(1) $$\begin{array}{c} Yield\;of\;essential\;oil\;\left(\%\right)=\frac{Weight\;of\;oil}{Weight\;of\;Eucalyptus\;leaves}*100\text{.} \end{array}$$

Chemical composition of the essential oil samples was determined by gas chromatography using an Agilent GC/MS (7890B) coupled to a quadripolar mass spectrometer (5977A) (Agilent Technologies, Palo Alto, CA, USA). A HP-SMS 5% Phenyl Methyl Silox capillary column (30 m × 250 *μ*m × 0.25 *μ*m) connected to a flame ionization detector (FID) was used with mobile phase flow (carrier gas: He) adjusted to 1.0 mL·min^−1^. The GC temperature program was 60°C at 4.0 min, then up to 240°C at a rate of 10°C per min, then to 300°C at a rate of 40°C per min (maintained for 5 min). The injector temperature was 280°C. Oil samples were diluted with methanol.

For GC/MS detection, an electron ionization system was used with ionization energy set at 70 eV and mass range *m*/*z* 40–300. The chemical components were detected and identified by comparison of the mass spectra using the NIST 5.01 Mass Spectral Library (Agilent P/N G1033A) and comparing retention times; homologous C8–C30 n-alkane series by determining the linear retention index (LRI) according to Van Den Dool and Kratz [20]. The relative amounts of individual components were performed by integrating the peak in the FID chromatogram and expressed as percent of area.

### 2.2. Animals and Treatments

Male *Swiss* (*Mus musculus*) mice aged 6–8 weeks (25–35 g), bred in the Unochapecó Animal Facility (Chapecó, Brazil), were used. The animals were kept in acrylic cages (28 × 12.5 × 19 cm) in groups of 4 mice per cage, fed with standard laboratory food (Biobase®) and water *ad libitum* in an air-conditioned room (22–24°C), 12 h light/dark cycle (lights on at 6 a.m.) and air humidity between 40 and 60%. Eight mice were used per experimental group and were set in the experimental room for at least 1 h prior to the experiments. The animals were fasted for 2 h (no water restriction) prior to oral administration. The tests were performed at 23 ± 1°C in the absence of stressful factors such as sounds, odors, and high luminosity. The doses of oils used in this study were chosen based on their 1,8-cineole content. Evidence shows that this compound presents anti-inflammatory activity at 40 mg/kg (p.o.) [21]. Therefore, the oils were administered at 50 mg/kg (p.o.). Only one species of *Eucalyptus* evaluated in this study does not present cineole in its composition; however, the same dose (50 mg/kg) was used. The solubilization of the oils was performed in saline solution with the aid of 1% polysorbate 80. Indomethacin (10 mg/kg, p.o.) was used as positive control [22, 23]. Animal care was conducted according to the ethical principles of the Directive 2010/63/EU [24] and Brazilian law no 11.794 (2008) [25]. The protocols were approved by the Ethics Committee of the Community University of Chapecó Region (approval number 005/2020).

### 2.3. Acetic Acid-Induced Abdominal Writhing Test

In this model, abdominal writhing was induced in mice according to the procedure described by Santos et al. [26] and adapted by our research group [27]. Contractions of the abdominal muscles together with the extension of one of the hind legs occur in response to an intraperitoneal (i.p.) injection of 0.6% acetic acid (10 mL/kg). The animals received vehicle, indomethacin (10 mg/kg - positive control [28]), or 50 mg/kg of the oils orally (p.o.) 1 h before the exposure to the injection of acetic acid. Before acetic acid administration, the animals remained in the transparent observation chamber for 20 min for recognition and adaptation to the site. Immediately after administration, abdominal contortions were quantified for 20 min. The oils that presented better effectiveness for the follow-up of the next behavioral trials were selected.

### 2.4. Formalin-Induced Paw Licking Test

This assay allows evaluating both neurogenic and inflammatory pain: direct stimulation of nociceptive fibers happens at first (1^st^ phase) and the inflammatory reaction (characterized by the release of inflammatory mediators) in a second moment (2^nd^ phase) [29]. The test was performed as previously described by Santos and Calixto [30] with minor modifications [27]: inflammation induction occurred by intraplantar (i.pl.) administration of 1% formalin (20 *μ*L) in the dorsal region of the right hind paw of mice. They were treated orally with vehicle, indomethacin (10 mg/kg - positive control [23]), or the oils under study 1 h before the exposure to formalin. Before formalin administration, the animals remained in the transparent observation chamber for 20 min for recognition and adaptation to the site. Immediately after formalin administration, mice were observed for 30 min: the number of paw elevations was quantified in the first 5 min and in the last 15 min. It was considered as paw elevation behavior any movement not associated with locomotion, ranging from a discrete elevation or contraction of the muscles of the animal's thigh to vigorous movement or licking and biting the paw.

### 2.5. Evaluation of Locomotor/Exploratory Activity

#### 2.5.1. Open Field Test

This assay was performed as described by Müller et al. [31] in order to verify a possible nonspecific effect of the oils on the spontaneous exploratory and locomotor activities of the animals, which may influence the results regarding the antinociceptive/anti-inflammatory activity. Mice were treated orally with vehicle or the oils that showed positive results in the nociception/inflammation tests. After 60 min, they were placed in the center of a black waterproofed MDF box (40 × 30 × 30 cm) with its bottom divided in 24 equal squares. After 5 min of familiarization, the number of crossings, rearing, and fecal bolus were quantified for 10 min.

#### 2.5.2. Rotarod Test

This model was carried out to verify the effects of the oils on motor coordination of the animals, as described by Neves et al. [32]. The rotarod device consisted of a cylinder (6 cm of diameter) rotating at 6.5 rpm. Initially, mice were trained to balance on the device for 5 min, including falls. Twenty-four hours later, the animals were submitted to a baseline responsiveness determination, when only the ones that presented a minimum continuous permanence time of 90 s were considered able to continue the test. Immediately after this session, the selected animals were treated orally with vehicle or the oils. One hour later, the test was performed and the longer permanence time (seconds) and number of falls within the observation period (5 min) were recorded.

#### 2.5.3. Acute Toxicity

The acute oral toxicity test was carried out following Guideline 423 from the Organization for Economic Cooperation and Development [33]. Nonpregnant female mice were treated with vehicle (*n* = 3, control group) or the *Eucalyptus* essential oils that showed significant results in the nociception/inflammation assays, at 2000 mg/kg (*n* = 6). Their behavior was observed individually with special attention in the first 4 h and 12 h after the treatment with regard to the following occurrence of: piloerection, palpebral ptosis, abdominal contortions, changes in locomotion, hypothermia, increased muscular tonus, shaking, posterior paws stoppage, salivation, bronchial secretion, convulsions, and deaths. The animals' body weight, food intake, and deaths were registered every 2 days. On the 15^th^ day after the treatment, they were euthanized by inhalation of isoflurane (dose of 10–12% in inhaled air) and confirmation by cervical displacement [34]; CONCEA [35] and the organs (liver, kidneys, adrenal glands, spleen, lungs, heart, and brain) relative weight (%) and macroscopic aspect were registered.

### 2.6. Antimicrobial Strains and Media

According to the method described by Gasparetto et al. [36], the antibacterial activity of the essential oils was evaluated against 1 Gram-positive (*Staphylococcus aureus* - ATCC 6538) and 1 Gram-negative (*Escherichia coli* - ATCC 25922) bacteria. The antifungal activity against the pathogenic fungi *Candida albicans* (ATCC 24433) was determined using the dilution technique. The Mueller–Hinton agar was the culture medium used for bacteria, which were incubated for 18 to 24 h at 35°C. For growing *C. albicans*, Sabouraud Dextrose 4% agar was used and the incubation conditions used were 24 to 48 h at 30°C. The strains were standard reference of the American Type Culture Collection (ATCC) (Rockville, MD, USA).

#### 2.6.1. Antimicrobial Assay

The minimum inhibitory concentration (MIC) of essential oils was determined by agar dilution assay according to the CLSI [37], with minor modifications [35]. The assay was carried out on slants (1 mL) against bacteria and *C. albicans*. A series of dilutions was prepared, concentrations ranging from 25,000 ppm-48 ppm. Afterwards, 1 *μ*L of inoculum suspension was added to each slant, except for the sterile control. A drug-free solution was also used as a blank control. Each assay was repeated 3 times. The bacterial strains were incubated at 35°C for 18 to 24 h and the yeast, at 30°C for 24 to 48 h. The MICs were visually recorded after 24 h for bacteria, 48 h for the yeast, and in accordance with control fungus growth for the remaining fungi [38]. For each concentration that did not show microbial growth in the MIC assay, the respective subcultures were performed in a medium without essential oil, followed by incubation and subsequent verification of the minimum microbicidal concentration (MMC) [39].

### 2.7. Statistical Analysis

Dataset normality was evaluated by the Shapiro−Wilk test. Considering that the results were parametric, the data of the behavioral assays and organs' weight were evaluated by one-way analysis of variance (ANOVA) followed by Bonferroni´s test. Body weight and food intake were evaluated by repeated measures two-way ANOVA (factor 1: day; factor 2: treatment) *post-hoc* Bonferroni test. The software GraphPad Prism 5.01 (GraphPad Software®, San Diego, California, USA) was used for the analysis of the results. Results were expressed as mean ± standard error of the mean (S.E.M.). *P* values less than 0.05 were considered significant.

## 3. Results and Discussion

### 3.1. Yield and Chemical Analyses of Essential Oils

The yield and compounds identified in the essential oils with their respective concentrations are shown in Table 1. The essential oil yield of the different varieties of *Eucalyptus* was in the range of 0.77-3.32%, in agreement with other works reported in the literature [40]. Knowledge of the essential oil yield is important for both commercial purposes and scientific research since it can affect the availability and economic viability of *Eucalyptus* essential oil production [41]. The plants that showed the greatest potential were Urcan and Grancan varieties, with yields of 3.32 and 2.30%, respectively.

It can also be seen from Table 1 that 1,8-cineole (eucalyptol) is present in all species studied except for *E. benthamii*. Note that the hybrid Urocam presented the highest concentration of this compound (82.18%), which makes this plant a potential source for this compound. Besides being a biologically active compound, it has a high therapeutic index and antiseptic properties [42]. According to Caceres et al. [43], eucalyptol inhibits the arachidonic acid pathway, thus inhibiting inflammatory mediators, and also 1,8-cineole and glucocorticoids have a mechanism of inhibiting inflammatory mediators in common.

Except for Grancam, *α*-pinene was also abundant in all *Eucalyptus* varieties studied. Such a compound has been considered an anti-inflammatory agent, mainly in osteoarthritis, besides presenting antioxidant and antiallergic activities and antimicrobial effectiveness against some strains of *S. aureus* and methicillin-resistant *Staphylococcus aureus*, among others [44, 45].

Another pinene that is part of the chemical composition of *Eucalyptus* essential oils is *β*-pinene, which presents biological activities such as anti-inflammatory, antidepressant, anxiolytic, hypotensive, antimicrobial, and myorelaxant, among others [46]. A high concentration of *p*-cymene in *Eucalyptus* essential oil was reported by Almeida et al. [47], with possible antiseptic activity that inhibited the growth of *S. aureus* and *E. coli*.

*γ*-Terpinene has been reported to present significant antimicrobial activity [48] and bacteriostatic activity against some microorganisms has been attributed to terpineol [49]. The *Benthamii* plant presented globulol as one of the major components (18.01%); this sesquiterpene has antifungal properties against a variety of fungal species as well as bacteria [50] and antimicrobial and antioxidant activities [51].

D-Limonene, a monoterpene found in several essential oils of aromatic species, was observed in 5 of the 7 *Eucalyptus* varieties studied, with concentrations ranging from 3.23 to 10.56%. D-limonene is a multifunctional compound with a potent therapeutic effect, widely used in the food and pharmaceutical industries [52, 53].

Overall, this study provides valuable information about the chemical composition of essential oils from the seven varieties of *Eucalyptus* cultivated in Brazil, which may explain the results of anti-inflammatory and antimicrobial activity obtained in this work and may have important applications in several industries, including pharmaceutical, cosmetic, and food.

### 3.2. Antinociceptive and Anti-Inflammatory Activities

#### 3.2.1. Acetic Acid-Induced Abdominal Writhing Test

**Figure 1:**
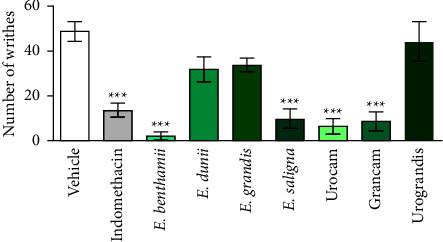
Effect of essential oils on leaves of *Eucalyptus* species obtained by supercritical CO_2_ on acetic acid-induced abdominal writhing test. Mice were treated with vehicle (0.9% NaCl plus 1% of tween 80, 10 ml/kg, p.o., negative control group), or indomethacin (10 mg/kg p.o., positive control group), or the essential oils of *E. benthamii*, *E. dunnii*, *E. grandis*, *E. saligna*, Urocam, Grancam, or Urograndis (50 mg/kg, p.o.), 1 h before i.p. acetic acid (0.6%) administration (*n* = 8). One-way ANOVA followed by Bonferroni´s test: ^*∗∗∗*^*P* < 0.001 compared to the vehicle-treated (negative control) group. Results expressed as mean ± S.E.M.

The acetic acid-induced abdominal writhing test showed that the positive control group (indomethacin at 10 mg/kg) and the groups treated with Urocam, Grancam, *E. benthamii,* and *E. saligna* (50 mg/kg, p.o.) presented a significant lower number of abdominal writhing when compared to the group treated with vehicle (*F* (8, 46) = 15.12, *P* < 0.0001) (Figure 1) (*F* (8, 63) = 15.12, *P* < 0.001).

It is known that 1,8-cineole presents anti-inflammatory and antinociceptive properties, while *α*-pinene shows anti-inflammatory and antiallergic activities [45, 54]. Results reported in this work agree with other studies that demonstrated that *Eucalyptus* essential oil reduced the number of abdominal writhing, similarly to the positive control used (indomethacin) [55], which may be related to the presence of 1,8-cineole and *α*-pinene in the essential oils.

Based on the chemical composition of the oils investigated, the following may be pointed out as the most efficient with regard to pharmacological effect: Urocam (82.15% of 1,8-cineole and 2.90% of *α*-pinene), Grancam (38.43% of 1,8-cineole and 35.92% of *α*-pinene), *E. benthamii* (satisfactory result with 41.20 of p-cymene and 17.39% of *α*-pinene only), and *E. saligna* (40.04% of 1,8-cineole and 33.32% of *α*-pinene). Considering the results obtained in the acetic-acid induced writhing test, these 4 plants - Urocam, Grancam, *E. benthamii,* and *E. saligna* - were selected to be used in the following assays.

#### 3.2.2. Formalin-Induced Paw Licking Test

In order to confirm the antinociceptive and/or anti-inflammatory activities of *Eucalyptus* essential oils, the ones that presented the best results on acetic acid-induced abdominal writhing assays were also tested in the formalin-induced paw licking test. This assay consists of two phases: the first one (0–5 min) corresponds to neurogenic pain, and the second one (15–30 min) is related to the inflammatory response. Neurogenic pain occurs in response to the direct stimulation of nociceptors by formalin, which results in the release of other mediators that perform local responses, causing vasodilation, activation of synaptic fibers, and leukocyte chemotaxis. In the inflammatory response induced by formalin, there is the release of inflammatory mediators that end up activating the inflammatory cascade and sensitizing nociceptive pathways [56]. Both phases can be inhibited by central-acting drugs, such as opioids, while NSAIDs are more effective in preventing inflammatory pain [57].

**Figure 2:**
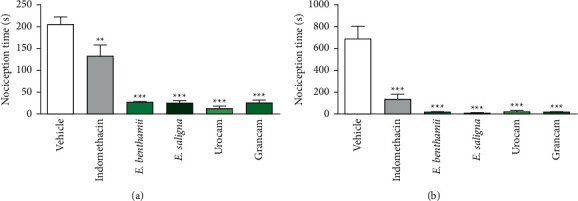
Effect of essential oils of leaves of *E. benthamii, E saligna,* Urocam, and Grancam, on formalin test: 1^st^ phase (a) and 2^nd^ phase (b). Mice were treated with vehicle (0.9% NaCl plus 1% of tween 80, 10 ml/kg, p.o., negative control group), or indomethacin (10 mg/kg p.o., positive control group), or the essential oils of *E. benthamii*, *E. saligna,* Urocam, and Grancam, (50 mg/kg, p.o.), 1 h before formalin (1%, i.pl.) administration (*n* = 8). One-way ANOVA followed by the Bonferroni´s test: ^*∗∗*^*P* < 0.01 and ^*∗∗∗*^*P* < 0.001 compared to the vehicle-treated (negative control) group. Results expressed as mean ± S.E.M.

Figure 2 represents the effects of *E. benthamii, E. saligna,* Urocam, and Grancam, tested in the formalin test. In the first phase (Figure 2(a)), the time that the animals spent licking the paw was significantly (*F* (5, 42) = 32.60, *P* < 0.0001) reduced by the essential oils of the four species when compared to the vehicle-treated group, as well as the animals treated with indomethacin.

The short nociception time in the first phase may indicate that the antinociceptive effect had the involvement of the opioid pathway, since it is known that some compounds of the oils, such as *β*-pinene and p-cymene, present an antinociceptive effect acting as a partial agonist of *μ*-opioid receptors [58, 59].

In the second phase of the test (Figure 2(b)), when the anti-inflammatory response of *Eucalyptus* essential oils tested were evaluated, and there was also a significant (*F* (5, 42) = 24.12, *P* < 000.1) reduction in paw licking time for all oils tested as well as indomethacin when compared to the vehicle-treated group.

The results of this test indicate that the essential oils of the leaves of the species *E. benthamii, E. saligna,* Urocam, and Grancam present antinociceptive action with central and peripheral effects by responding to neurogenic and inflammatory pain caused by formalin injection. This is probably related to the major compounds of the essential oils studied, 1,8- cineole, p-cymene, and *α*-pinene, which present anti-inflammatory and antinociceptive activities [43, 59].

The compound 1,8-cineole inhibits cyclooxygenase enzymes, resulting in the decrease on the production of TNF-*α*, leukotriene B4, and thromboxane A2, and it is also a potent inhibitor of cytokines [43]. The study of Santos and Rao [60] corroborates such facts: in the carrageenan test, which induces paw edema, 1,8-cineole at 400 mg/kg (p.o.) inhibited the edema, and, according to the authors, it indicates a decrease in the production of inflammatory mediators. In addition, there was also a reduction in the nociception time in both phases of the formalin test at the same dose of 1,8-cineole.

*α*-Pinene, when investigated through the carrageenan-induced paw edema test, inhibited the edema and also decreased leukocyte migration at 200 and 400 mg/kg [45]. In the study by Lee et al. [55], *Eucalyptus* essential oil at 45 mg/kg p.o. significantly reduced the nociception time only in the 2^nd^ phase of the formalin test.

### 3.3. Evaluation of Locomotor/Exploratory Activity

#### 3.3.1. Open Field Test

The open field test was carried out to exclude the possibility that the results of the behavioral assays of nociception and inflammation were related to a nonspecific effect of the essential oils of *E. benthamii*, *E. saligna,* Urocam, and Grancam on spontaneous exploratory and locomotor activities of the animals.

**Figure 3:**
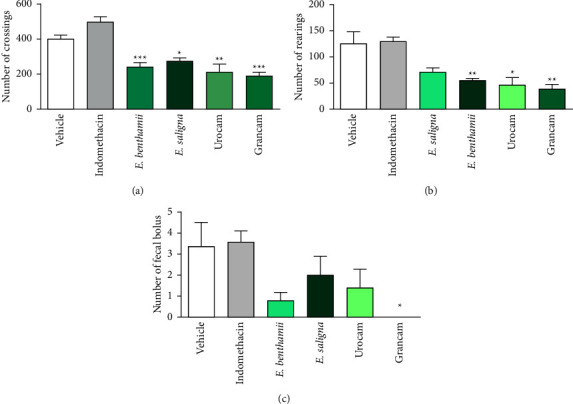
Effect of essential oils on leaves of *E. benthamii, E saligna,* Urocam, and Grancam on the open field test: number of crossings (a), rearing (b) and fecal bolus (c) expelled during the session. Mice were treated with vehicle (0.9% NaCl plus 1% of tween 80, 10 ml/kg, p.o., negative control group), or indomethacin (10 mg/kg p.o., positive control group) or the essential oils of *E. benthamii*, *E. saligna,* Urocam, or Grancam (50 mg/kg, p.o.), 1 h before the exposure to the apparatus (*n* = 8). One-way ANOVA followed by Bonferroni´s test: ^*∗*^*P* < 0.05, ^*∗∗*^*P* < 0.01, and ^*∗∗∗*^*P* < 0.001 compared to the vehicle-treated (negative control) group. Results expressed as mean ± S.E.M.

Figure 3 illustrates the number of crossings of the animals treated with the essential oils. There was a significant reduction in the number of crossings (Figure 3(a)) of animals treated with all oils studied (*F* (5, 42) = 18.03, *P* < 000.1). Also, according to Figure 3(b), mice treated with *E. benthamii*, Urocam, and Grancam (*F* (5, 42) = 6.53, *P* = 0.0004) showed a significant reduction in the number of rearing compared to the vehicle-treated group. On the one hand, substances that increase the number of crossings and rearing are considered as stimulants [61]. On the other hand, a reduction in these parameters, as caused by the oils under study, is indicative of central depressant effects [61]. In line with our observations, Santos and Rao [60] demonstrated that 1,8-cineole caused a reduction in the locomotion of the animals in the open field test, hence suggesting a sedative effect. Furthermore, it is known that the number of crossings can be affected by central-acting drugs or peripheral muscle relaxers [62]; the number of rearing assesses the degree of sedation and anxiety and can be altered by drugs with anxiolytic/anxiogenic activity; and the number of fecal bolus can be altered by anxiolytic, anxiogenic, spasmolytic, or spasmogenic drugs [63].

In Figure 3(c), the number of fecal bolus is depicted: the animals treated with Grancam showed a significant (*F* (5, 42) = 2.767, *P* = 0.038) reduction in the number of fecal bolus expelled compared to the group treated with vehicle. The reduction in the number of fecal bolus may be related to the involvement of the opioid receptors in the mechanism of action of this oil since opioid drugs present regularly reported peripheral side effects such as constipation, urinary retention, and pruritus [64].

The satisfactory antinociceptive activity as well as the constipation and sedative effects are qualities of opioid drugs [65]. Indeed, the chemical composition of the oils tested demonstrated the presence of some compounds that act as opioid agonists, such as *β*-pinene [58]. Therefore, it can be suggested that the oils tested are very efficient in the pain management process, perhaps with theinvolvement of the opioid pathway. However, their mechanism of antinociceptive action must be elucidated.

#### 3.3.2. Rotarod Test

Considering that a sedative effect of the oils was detected in the open field test, the rotarod test was performed to verify the effect of the oils on the integrity of the motor coordination of mice.

Table 2 shows the results of the permanence time and number of falls of the animals treated with the oils in the rotarod test. There was no significant difference between the parameters evaluated between the groups. Thus, these results indicate that the essential oils of *E. benthamii*, *E. saligna,* Urocam, and Grancam did not alter the integrity of the motor coordination of the animals. Sobreira et al. [66] also noted that 1,8-cineole, the major component of *Eucalyptus* essential oil, did not cause changes in the motor coordination of the animals in the rotarod test, therefore in agreement with the results of this study.

### 3.4. Acute Toxicity

**Figure 4:**
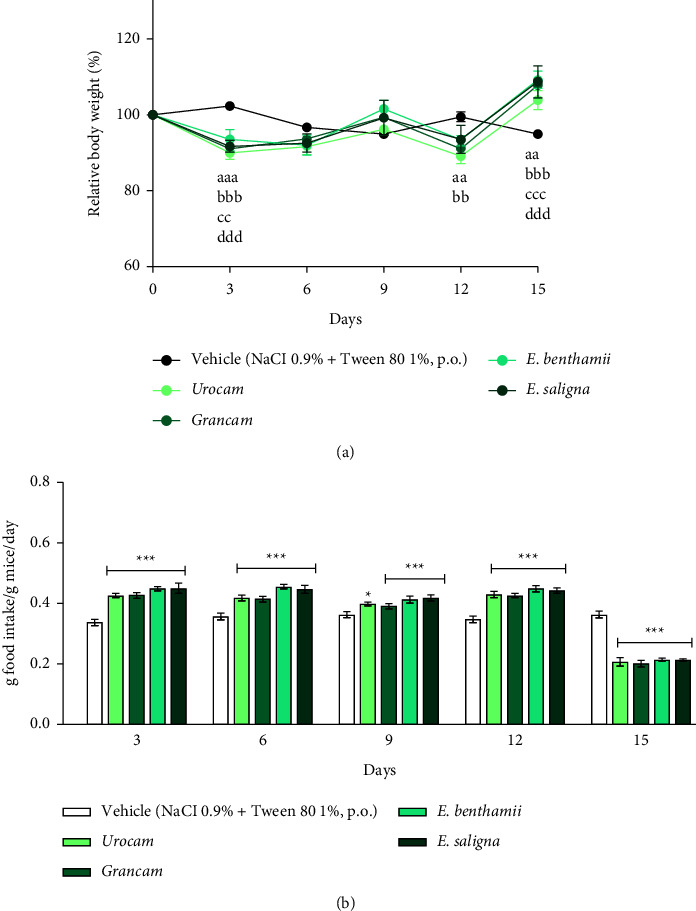
Acute oral toxicity (2000 mg/kg, p.o.) on relative body weight (%) (a) on female mice food intake (g food intake/g mice/day) and (b). Mice were treated with vehicle (0.9% NaCl plus 1% of tween 80, 10 ml/kg, p.o., negative control group, *n* = 3) or the essential oils of Urocam, Grancam, *E. benthamii*, or *E. saligna* (2000 mg/kg, p.o., *n* = 6). Repeated measures two-way ANOVA followed by the Bonferroni test: (a) compared to the vehicle-treated (negative control) group, Urocam: ^aa^*P* < 0.01, ^aaa^*P* < 0.001; Grancam: ^bb^*P* < 0.01, ^bbb^*P* < 0.001; *E. benthamii*: ^cc^*P* < 0.01, ^ccc^*P* < 0.001; *E. saligna*: ^ddd^*P* < 0.001. (b) ^*∗*^*P* < 0.05, and ^*∗∗∗*^*P* < 0.001 compared to the vehicle-treated (negative control) group at the same day. Results expressed as mean ± S.E.M.

In the acute toxicity assay, the selected essential oils were administered at a single dose of 2000 mg/kg (p.o.). Figures 4(a) and 4(b) present the relative body weight and food intake of the animals, respectively.

A two-way RM ANOVA revealed a significant effect of day (*F* (5, 125) = 56.77 , *P* < 0.0001])and a significant interaction between factors (day x treatment) (*F* (20, 125) = 7.943, *P* < 0.0001) on the relative body weight of animals. No effect of treatment was detected on mice relative body weight (*F* (4, 25) = 0.7158, *P* = 0.589).

Regarding food intake, a two-way RM ANOVA revealed significant effect of day (*F* (4, 100) = 656.8 , *P* < 0.0001), treatment (*F* (4, 25) = 4.94, *P* = 0.0045) and a significant interaction between factors (day × treatment) (*F* (16, 100) = 49.20, *P* < 0.0001). *Posthoc* analysis revealed that on the 3^rd^ day of the experiment, a significant decrease in body weight (*P* < 0.01 for *E. benthamii* and *P* < 0.001 for the other ones) and a significant increase in food intake (*P* < 0.001) were observed in the groups treated with *Eucalyptus* species when compared to the vehicle-treated group. At the last experimental day, the animals treated with *Eucalyptus* ingested significantly (*P* < 0.001) less food and their body weight significantly increased (*P* < 0.01 for *Urocam* and *P* < 0.001 for the other ones) in comparison with the vehicle-treated group.

These data suggest that *Eucalyptus* essential oils present a metabolic side effect. On the 15^th^ day of observation, when the animals treated with the oils ingested less food and recovered their body weight, it was likely that the effect of *Eucalyptus* essential oils on metabolism had stopped [66, 67]. Some tests on the toxicity of *Eucalyptus* essential oils in rats showed that their median lethal dose (LD50) was 4,440 mg/kg, and their major oil compound, 1,8-cineole, had a LD50 of 2,480 mg/kg. Tests on mammals such as koalas demonstrated a certain toxicity of the *Eucalyptus* oils tested [68]. In addition, there was no difference in both the body weight of animals and organs compared to the vehicle-treated group when isolated 1,8-cineole was tested [69].

**Figure 5:**
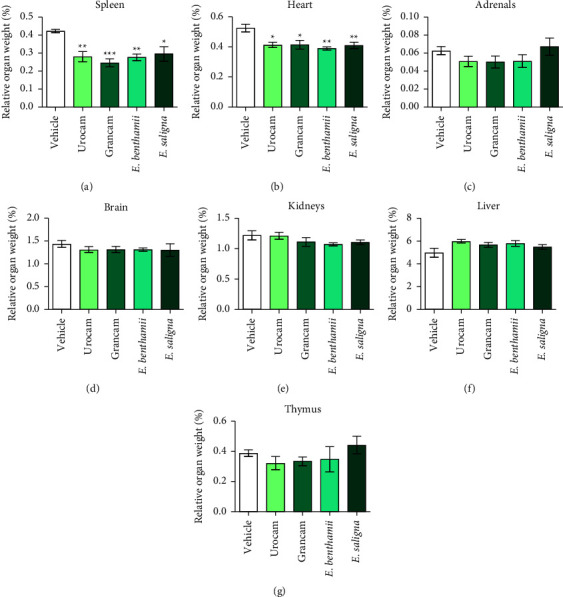
Acute oral toxicity (2000 mg/kg, p.o.) on relative organs weight (%): spleen (a), heart (b), adrenals (c), brain (d), kidneys (e), liver (f), and thymus (g). Mice were treated with vehicle (0.9% NaCl plus 1% of tween 80, 10 ml/kg, p.o., negative control group, *n* = 3) or the essential oils of Urocam, Grancam, *E. benthamii*, or *E. saligna* (2000 mg/kg, p.o., *n* = 6). One-way ANOVA followed by Bonferroni test: ^*∗*^*P* < 0.05, ^*∗∗*^*P* < 0.01, and ^*∗∗∗*^*P* < 0.001 compared to the vehicle-treated (negative control) group at the same day. Results expressed as mean ± S.E.M.

The effect of essential oils on the weight of animals' organs is shown in Figure 5. The treatments with all studied essential oils significantly reduced the relative weight of spleen (*F* (4, 25) = 7.024, *P* = 0.0006) (Figure 5(a)) and heart (*F* (4, 25) = 6.205, *P* = 0.0013) (Figure 5(b)). There were no effects of the essential oils on the relative weight of mice's adrenals (*F* (4, 25) = 1.347, *P* = 0.280) (Figure 5(c)), brain (*F* (4, 25) = 0.466, *P* = 0.760) (Figure 5(d)), kidneys (*F* (4, 25) = 1.397, *P* = 0.234) (Figure 5(e)), liver (*F* (4, 25) = 2.202, *P* = 0.097) (Figure 5(f)), and thymus (*F* (4, 25) = 0.867, *P* = 0.497) (Figure 5(g)).

Unlike the results found here, there are no reports of splenic and cardiac toxicity related to *Eucalyptus* essential oils in the literature. Furthermore, the reduction in the proportion of cardiac weight has also not been described for *Eucalyptus* species and; therefore, there is no conclusive cause of the reason why this effect was observed.

The results of acute toxicity need to be confirmed in repeated dose toxicity studies, with the use of extrapolated working doses, as recommended by the OECD [33]. Anyway, the treatment with the *Eucalyptus* oils studied did not cause any toxicity sign or death during the study period. Thus, according to Guideline 423 [33], they can be classified in category 5 of the Globally Harmonized System (LD50 is > 2000 mg/kg-5000 mg/kg for nonvulnerable populations).

### 3.5. Antimicrobial Assay

The seven oils demonstrated activity against *S. aureus* (Gram-positive bacteria) and *E. coli* (Gram-negative bacteria) (Table 3). Besides, they also presented activity against the fungus *C. albicans*, as shown in Table 4. Estanislau et al. [70] evaluated the antimicrobial activity of 5 *Eucalyptus* species and also obtained promising results against *S. aureus* and *E. coli* for all oils tested. However, the technique used by them was the disk diffusion method; therefore. it is not possible to advance in this discussion.

Additionally, *E. dunnii* and *Grancam* had the best performance in MIC and MMC assays for the two bacteria under study. The 2 species have different concentrations of active substances: *E. dunnii* presents 8.55% *α*-pinene and 63.01% of 1,8-cineole, while *Grancam* presents 35.92% of *α*-pinene and 38.43% of 1,8-cineole. These compounds are responsible for the main antimicrobial effectiveness against some strains of *S. aureus* and have high therapeutic indexes [42, 44]. *E. dunnii* and Grancam present 8.42% and 2.62% of terpineol, respectively, which is responsible for bacteriostatic action [49].

The compound D-limonene is present in *E. dunnii* (3.52%) and in *Grancam* (10.52%) and possesses antimicrobial activity [71]. *β*-pinene also presents antimicrobial activity and is part of *Grancam* (2.75%) essential oils [46].

Furthermore, the essential oil of the studied varieties has other components in lower concentrations in its composition. According to Chorianopoulos et al. [72], antimicrobial activity occurs not only through the presence of major compounds but also by the influence of other components at lower concentrations that can cause synergistic, additive, or antagonistic interactions.

The antifungal activity of the oils was slightly more promising in view of the results of MIC and MMC. Cimanga et al. [73] compared the antimicrobial activity of the essential oils of some *Eucalyptus* species. The major compounds (such as 1,8-cineole and *α*-pinene) were also tested isolated and had less activity than the essential oil, stating that the combination of compounds is important for the antimicrobial activity of *Eucalyptus* oil [73], thus showing that it is not possible to compare the compounds of the oils isolated.

The mechanism of inhibitory/microbicidal action of the essential oils may be related to their characteristics, such as hydrophobicity, which allows their interaction with cellular lipid structures, promoting increased permeability and consequent extensive efflux of electrolytes, which play an essential role in cell homeostasis [74].

According to Burt [75], the difficulty in comparing the results obtained by several authors is often mentioned as a problem faced in the study of the antimicrobial activity of essential oils and plant-derived products, since there are variations in the methods used.

Results reported in this work reinforce the information that *Eucalyptus* essential oils have antimicrobial activity, especially antifungal, with the advantage of being a natural product. According to Rahman and Kang [76], the risk that pathogenic microorganisms will develop resistance to essential oils is very low, since they contain a mixture of antimicrobial substances that act through various mechanisms. These characteristics represent an advantage of essential oils as antimicrobial drugs, which can be promising for applications in different areas.

## 4. Conclusion

In this study, the great chemical variability that exists between the essential oils of the various *Eucalyptus* species was demonstrated, highlighting Urocam, Grancam, and E. *dunii*, which obtained the highest oil yield with high concentrations of 1.8 cineole and a-pinene. In addition, the essential oils of Urocam, Grancam, *E. benthamii,* and *E. saligna* have antinociceptive and anti-inflammatory activities and are devoid of acute toxicity. Also, the seven varieties of *Eucalyptus* studied are active against *S. aureus*, *E. coli,* and *C. albicans*. Finally, the *Eucalyptus* essential oils represent an important source of antinociceptive, anti-inflammatory, and antimicrobial compounds, such as 1,8-cineole and *α*-pinene. Future studies on the elucidation of the mechanism of the antinociceptive action of the oils are required.

## Figures and Tables

**Table 1:** Chemical composition and yield (%) of essential oil from different *Eucalyptus* varieties.

	RT	RI-N	RI-E	*E. benthamii*	Grancam	*E. dunnii*	*E. grandis*	*E. saligna*	Urocam	Urograndis
*α*-pinene	4.24	937	937	17.39	35.92	8.55	30.54	33.32	2.90	26.44
Camphene	4.48	943	953		0.87		3.00	0.77		0.95
*β*-pinene	5.10	943	993		2.75					
p-cymene	5.71	1036	1033	41.20		0.79	1.93	3.60	0.85	28.87
*β*-terpinyl acetate	5.78	1348	1032					3.70		
D-limonene	5.78	1018	1032		10.56	3.52	3.74		5.17	3.23
1.8-cineole	5.86	1023	1036		38.43	63.01	8.20	40.04	82.15	11.74
*γ*-terpinene	6.31	998	961	4.19						5.67
Fenchol	7.42	1125	1121		0.96		3.68			0.83
*α*-Campholenal	7.61	1128	1131		0.44		2.41	2.23		0.60
L-pinocarveol	7.97	1135	1150		2.46		2.93	1.63		0.97
Pinocarvone	8.35	1164	1170		1.10			0.63		0.38
(−)-borneol	8.43	1173	1175		1.39		9.73			
Camphol	8.44	1148	1175				0.69	2.21		2.02
4-terpinenol	8.63	1137	1185	2.51						0.94
*α*-terpineol	8.91	1172	1200		2.62	8.42	9.38	3.76	1.36	3.44
*α*-terpineol acetate	11.87	1333	1356						6.45	3.60
Caryophyllene	13.27	1494	1430							1.30
Aromandendrene	13.59	1439	1466	11.45		3.04				
Cadina-1(10).4-diene	15.03	1537	1532							1.24
Epiglobulol	15.72	1530	1472	4.17		1.91				
Spathulenol	16.03	1536	1490				1.29	4.40		2.33
Globulol	16.15	1578	1497	18.01		7.40		2.40		1.39
Himbaccol	16.29	1530	1507							0.77
Viridiflorol	16.30	1554	1507			1.48				
Total				98.92	97.50	98.12	73.33	98.69	98.88	96.71

Oil yield (%)				1.72 (±0.19)	2.30 (±0.20)	1.56 (±0.15)	0.77 (±0.04)	0.85 (±0.09)	3.32 (±0.21)	1.35 (±0.14)

LR-N-bookstore linear retention index national institute of standards and technology, RT-retention time, and RI-E-experimental retention index.

**Table 2:** Effects of *Eucalyptus* essential oils on the motor coordination of mice, evaluated by the rotarod test. Mice were treated with vehicle (0.9% NaCl plus 1% of tween 80, 10 ml/kg, p.o., negative control group) or indomethacin (10 mg/kg p.o., positive control group) or the essential oils of *E. benthamii*, *E. saligna,* Urocam, or Grancam (50 mg/kg, p.o.) (*n* = 8).

Treatment	Permanence time (s)	Number of falls
Vehicle	299.3 ± 0.75	0.12 ± 0.12
Indomethacin	299.3 ± 0.49	0.25 ± 0.16
*E. benthamii*	273.7 ± 1.58	0.12 ± 0.54
*E. saligna*	255.7 ± 2.70	0.37 ± 0.79
Urocam	300.0	0.0
Grancam	300.0	0.0

One-way ANOVA. Results expressed as mean ± S.E.M.

**Table 3:** Antibacterial activity of *Eucalyptus* essential oils against *S. aureus* and *E. coli*.

Minimum inhibitory concentration (MIC)/minimum microbicidal concentration (MMC)		
Essential oil	Gram-positive bacteria	Gram-negative bacteria
	*S. aureus* (%)	*E. coli* (%)
*E. benthamii*	0.625/1.25	1.25/2.5
*E. dunii*	0.312/0.625	0.312/0.625
*E. grandis*	0.625/1.25	1.25/1.25
*E. saligna*	0.625/1.25	1.25/2.5
Urocam	1.25/2.5	1.25/1.25
Grancam	0.312/0.625	0.625/1.25
Urograndis	0.625/1.25	1.25/1.25

**Table 4:** Antibacterial activity of Eucalyptus essential oils against C. albicans.

Minimum inhibitory concentration (MIC)/minimum microbicidal concentration (MMC)	
Essential oil	*C. albicans* (%)
*E. benthamii*	0.156/0.312
*E. dunii*	0.156/0.312
*E. grandis*	0.625/0.625
*E. saligna*	0.312/0.312
Urocam	1.250/1.250
Grancam	0.625/1.250
Urograndis	0.312/0.625

## Data Availability

The data used to support the findings of this study are available from the corresponding author upon request.
